# Strengthening the policy framework to resolve lax implementation of the Baltic Sea Action Plan for agriculture

**DOI:** 10.1007/s13280-021-01573-3

**Published:** 2021-06-18

**Authors:** Mark V. Brady, Mikael Skou Andersen, Anna Andersson, Emils Kilis, Sanna-Riikka Saarela, Martin Hvarregaard Thorsøe

**Affiliations:** 1 Department of Economics, AgriFood Economics Centre, Swedish University of Agricultural Sciences (SLU), Box 730, 220 07 Lund, Sweden; 2 Centre for Environmental and Climate Science (CEC), Lund University, Box 188, 221 00 Lund, Sweden; 3 Department of Environmental Science, Aarhus University, Nordre Ringgade 1, 8000 Århus C, Denmark; 4 Baltic Studies Centre, Kokneses prospekts 26-2, Riga, 1014 Latvia; 5 Finnish Environment Institute (SYKE), Latokartanonkaari 11, 00790 Helsinki, Finland; 6 Department of Agroecology, Aarhus University, Blichers Alle 20, Foulum, 8830 Tjele, Denmark

**Keywords:** Agri-environment, CAP, Governance, HELCOM, Nutrient, Rural development

## Abstract

In this perspective article, we provide recommendations for strengthening the policy framework for protecting the Baltic Sea from agricultural nutrient pollution. The most striking weakness is the lax implementation of prescribed abatement measures, particularly concerning manure management, in most countries. Institutions of the EU should also be leveraged for achieving Baltic Sea Action Plan (BSAP) goals. In contrast to the Helsinki Convention, the European Union has economic, political and legal mandates to further implementation and compliance. Equally important is the need for strengthening of local institutions, particularly Water Boards and independent agricultural advisory services in the eastern Baltic Sea Region countries. There is also an urgent need for implementation of voluntary land-use measures where EU funding available to farmers is more broadly and effectively used by providing it on the basis of estimated abatement performance, which can be realized through modelling. The enormous potential for funding performance-based schemes, manure management infrastructure and advisory services through the EU’s Common Agricultural Policy are currently underutilized.

**Supplementary Information:**

he online version contains supplementary material available at 10.1007/s13280-021-01573-3.

## Introduction

Agricultural nutrient pollution of the Baltic Sea has been reduced over recent decades, but remains too high for achieving mutually agreed upon water quality and ecological goals (HELCOM 2018a). Nitrogen (N) and phosphorus (P) play key roles in the Sea’s continuing degradation; diffuse nutrient emissions from agriculture accumulate through riverine and atmospheric transports and mix over long distances, thereby contributing to widespread eutrophication (HELCOM 2018b). Cyanobacterial summer blooms and extensive bottom water hypoxia are now more pronounced than ever (Carstensen et al. 2014; ESA 2020).

Following HELCOM’s 1990 Rønneby declaration, abatement efforts focussed on identifying and cleaning up pollution ‘hot-spots’ through treatment of wastewater discharges from industry and conurbations. From 1998, agriculture became subject to concerted efforts to reduce nutrient emissions, as specified in amendments to the *Convention* on the Protection of the Marine Environment of the Baltic Sea Area. On account of the EU acceding to the Convention in 1994, there is substantial overlap between the EU’s Nitrates Directive and the measures prescribed to control nutrients from agriculture in Annex III, Part 2 of the Convention. To attain good ecological status in the open Sea, the 2013 Baltic Sea Action Plan (BSAP) aims to reduce the annual inputs of N by 13% (118,134 tonnes/year) and P by 41% (15,178 tonnes/year) compared to the reference period, 1997–2003.^1^ The BSAP also reflects the commitment of EU member states to implement the Water Framework Directive and Marine Strategy Framework Directive (Nilsson and Bohman 2015). By 2007 an extended list of agricultural abatement measures was agreed upon (reproduced in Table S1); however, unlike the prescribed measures of the Convention Annex these are not legally binding.

Three recent synthesis articles on governance structures and policy performance in the Baltic Sea region identify fundamental weaknesses in the current policy framework for controlling agricultural nutrient emissions to the Sea. Thorsøe et al. (2021) find that there has been lax implementation of the prescribed measures in most of the nine signatory countries. The omissions reflect insufficient capacities in government institutions to formalize the prescribed measures into national legislations, and to coherently integrate these with agricultural policy while empowering stakeholders through local institutions (Andersen et al. 2021). Further, unsettled issues about the cost-effectiveness of country-specific abatement targets and the fairness of the implied distribution of abatement costs likely undermine the determination of some countries for fulfilling their obligations (Andersson et al. 2021). Together, these weaknesses in the policy framework are a major hinder to achieving BSAP goals, because agriculture continues to be the major anthropogenic source of N and P emissions to the Sea (HELCOM 2018b).

The aim of this perspective article is to recommend actions for strengthening the policy framework for protecting the Baltic Sea from agricultural nutrient emissions. Our arguments are based on the weaknesses identified in the current framework in the three synthesis articles cited above and complemented by additional policy-relevant research literature, thereby helping to bridge the gap between Baltic Sea policy research and its implementation.

In their recent perspective article, Ollikainen et al. (2019) argue that there is a strategic need to move towards a Baltic Sea socioeconomic plan, as well as to bridge the growing gap between state-of-the-art water policy instruments and the existing policy framework for protecting the Baltic Sea. We see our paper as an important complement to their article, in particular, we argue there is a fundamental need to first bridge the gap between the basic abatement measures that have been agreed upon—to ensure minimum nutrient management standards—and what is actually occurring in most countries, as well as securing the necessary institutional support, both supra-nationally and locally, to ensure compliance with agreements and support introduction of state-of-the-art policy instruments; including novel ways to generate evidence of farmers’ abatement results, as a basis for calculating economic incentives to improve nutrient utilization and increase abatement. In this regard, we also identify greater potential to resolve these problems through institutions of the EU, particularly utilizing the reformed post-2020 Common Agricultural Policy (CAP) in combination with local institutions, to complement the inherent limitations of HELCOM and its BSAP, as witnessed below, by decades of lax implementation of agreed-upon agricultural abatement measures.

We structure this article around what we consider to be the three pillars of the policy framework for guiding farmers’ nutrient management in the Baltic Sea Region (BSR): (i) minimum nutrient management standards or measures; (ii) voluntary payment schemes for additional abatement measures; and (iii) the institutional framework characterizing each country, particularly the interdependent roles of the EU and local institutions to empower stakeholders.

## Sources of and preconditions for controlling agricultural emissions

**Fig. 1:**
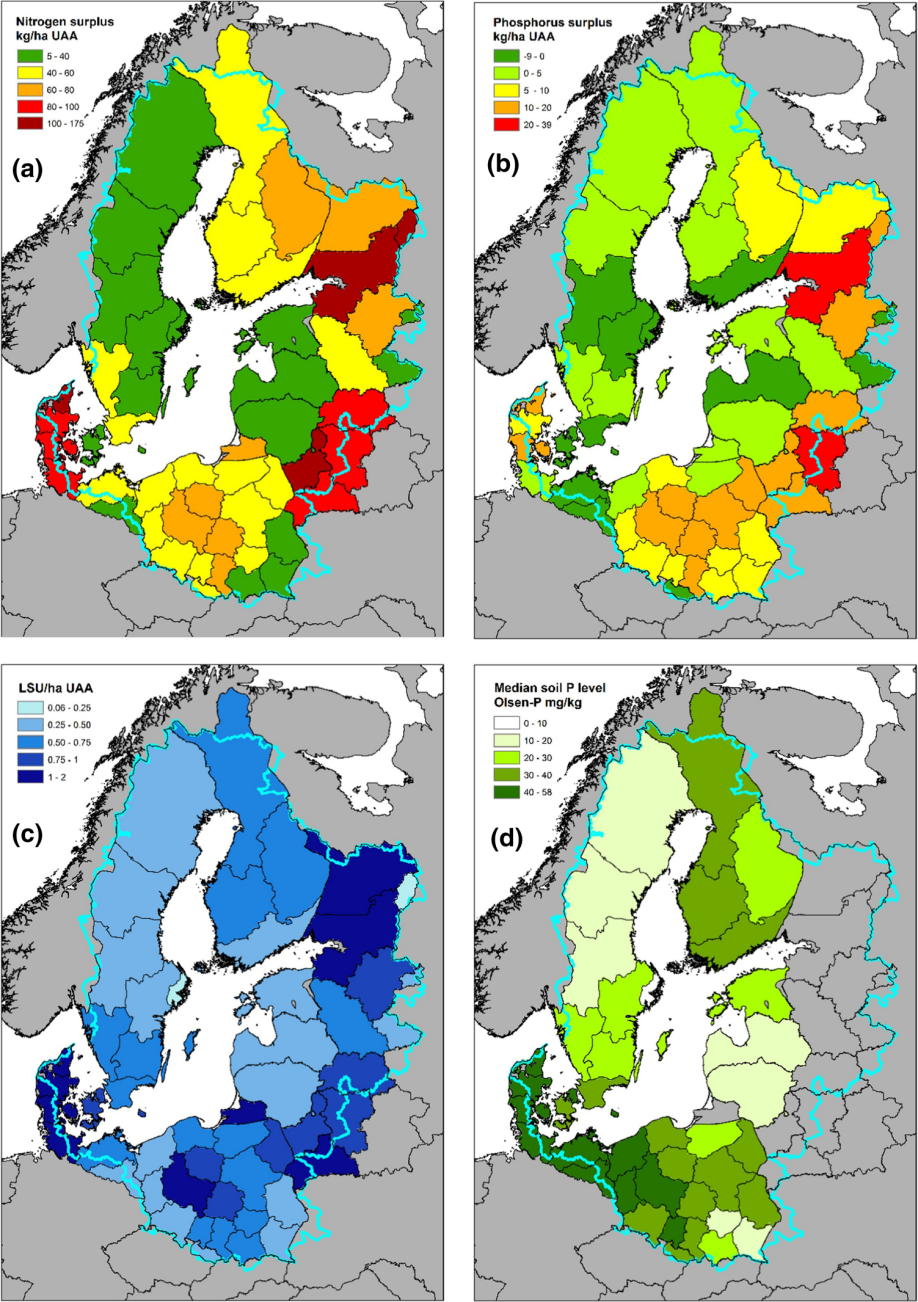
Important determinants of high rates of agricultural nutrient emissions to the Baltic Sea are spatial variations in **a** nitrogen surpluses, **b** phosphorous surpluses, **c** livestock densities and **d** soil phosphorous stocks Source: reproduced from Svanbäck et al. (2019) with permission of Elsevier

There exists substantial spatial variability in rates of nutrient emissions from agricultural land in the BSR as well as the preconditions for farming, and hence for controlling these emissions (HELCOM 2018b). Applying nutrients in excess of crop needs results in nutrient surpluses and increasing rates of nutrient emissions, while keeping in mind that emission rates can be moderated or exacerbated by soil properties, retention processes and climate. Large surpluses emerge in the BSR where the quantities of nutrients being applied to agricultural land in the form of mineral or manure fertilizers far exceed the quantities leaving the land in agricultural outputs such as food (Fig. 1a, b). In particular, high livestock concentrations in many areas are resulting in extreme surpluses, because large quantities of nutrients enter farms in purchased feed (Fig. 1c). An additional characteristic of phosphorous is that it builds up as a stock in soils because it binds to soil particles. Historical applications of manure have therefore resulted in relatively high soil P stocks in regions that have or have had high livestock densities (Fig. 1d), compounding the potential for high P emissions through runoff. Consequently, high livestock densities and associated manure surpluses are fundamental drivers of excessive agricultural nutrient emissions to the Sea.

That manure-nutrient surpluses accumulate in extremes in some areas, despite their potential for replacing costly mineral fertilizers and improving soil fertility, is explained by the high cost (given current market conditions) of transporting manure more than a few kilometres or processing it to reduce water content. In fact it is economically rational for farmers to generously or “over” apply manure to fields close to livestock facilities, and to only apply mineral fertilizers on fields distant from facilities (Lötjönen et al. 2020). Additionally, since it is far easier to apply mineral fertilizers according to crop needs over the growing cycle and within fields, nutrient surpluses tend to be relatively low in specialized arable cropping regions. For instance, mineral fertilizer can be applied in growing crops or in slow-release forms, and applied with precision technologies that adjust application rates to crop needs down to square decimetres within fields based on soil-sample data, advanced plant sensors and GPS, so-called precision agriculture (Lindblom et al. 2017), which is less feasible for manure application (SEGES, 2018). Still, it is not possible for crops to take up all applied mineral fertilizer, rates of which are also heavily influenced by random weather events. Moreover, crops can at best only take up 60–80% of the N applied in manure and as the contents are uncertain, farmers in livestock dense regions regularly apply mineral fertilizers to compensate for the relative limitations of manure fertilization (Webb et al. 2013). Accordingly, applications of mineral fertilizers can result in nutrient surpluses even in specialized arable cropping regions and compound nutrient surpluses in regions with livestock.

Nutrient surpluses also vary between regions with similar livestock densities and production conditions due to variations in management practices. This emphasizes the importance of nutrient bookkeeping to keep track of nutrient balances and the potential for excess application of mineral fertilizers. Accordingly, farmer training, as well as effective manure storage and spreading technologies are first steps for controlling emissions.

Large differences exist across the BSR in agricultural structure and farm profitability, as well as the relative importance of agriculture for national economies (Table 1). In terms of scale, Poland dominates all other countries, accounting for almost 50% of farmland and 75% of farms in the Baltic Sea catchment. Average farm incomes in Poland though are low, as well as in Latvia and Lithuania. In spite of this, agriculture is relatively important for the economies (GDP) and labour markets in these countries. Even large numbers of semi-subsistence farms (i.e. farms consuming more than 50% of their own output) can be found in Poland and the three Baltic states (Estonia, Latvia and Lithuania) (Žakevičiūtė 2019). However, farm incomes are also highly variable within these countries due to the co-existence of very large corporate farms, which are relatively few but manage a large proportion of the countries’ agricultural areas. In contrast, the value of average standard farm output is relatively high in Sweden and Finland, and clearly highest in Denmark and Germany (i.e. the two German regions in the catchment). These productivity differences reflect not only crop-yield potentials, but also high livestock densities, particularly in Denmark and Germany. Clearly, political priorities and the feasibility of individual farmers to bare abatement costs will differ among the BSR countries.

**Table 1:** Structural characteristics of agriculture, economies and public support to agriculture in Baltic Sea Region countries

	Germany^a^	Denmark	Sweden	Finland	Estonia	Latvia	Lithuania	Poland	Sum or average BSR	Belarus	Russia
*Structure*											
Number of farms	18 850	35 050	62 940	49 710	16 700	69 930	150 320	1 410 700	1 814 200		
Average farm size (ha)	122	75	48	45	60	28	19	10	17		
Agricultural land area (‘000 ha)	2295	2615	3013	2233	995	1931	2925	14 406	30 412	3969	913
Holdings with manure storage facilities (%)	77%	69%	95%	90%	31%	47%	11%	53%	52%		
Agricultural area of total land cover (%)	60%	62%	7%	8%	23%	31%	47%	47%	41%		
Permanent grassland (%)	0%	9%	15%	1%	31%	33%	26%	22%	18%		
*Consumption*											
Fertilizer (kgN/ha)	140	107	77	64	54	60	78	106	96		
Fertilizer (KgP/ha)	9	9	6	6	6	9	11	14	11		
*Livestock*											
Density (Livestock unit/ha UAA)	1.09	1.58	0.56	0.49	0.28	0.26	0.29	0.66	0.68	0.92	1.17
Share of farms with > 100 LSU (%)	27%	11%	6%	5%	2%	1%	0%	1%	5%		6%
Share of livestock on farms > 100 LSU (%)	89%	94%	70%	57%	77%	51%	46%	41%	56%		91%
*Economic*											
Standard output (€/ha)	2674	3405	818	631	665	403	703	1540	1466		
Standard output (€/farm)	178 361	287 088	81 962	70 702	47 997	17 465	14 810	17 726	63 953		
Farms consuming > 50% of own output (%)	0	0	0	0	29%	39%	45%	18%	19%		
Agricultural output (% GDP)	1.5%	3.2%	1.1%	1.5%	3.1%	4.5%	5.5%	5.0%	3.2%		
Employment in agriculture (% workforce)	1.3%	2.5%	1.9%	3.9%	3.9%	7.7%	8.0%	10.5%	5.0%		
Public financial support^b^											
Farmers (€/ha)	503	489	375	583	350	308	368	412	422		
Farmers (€/farm)	32 053	38 021	18 288	27 254	23 926	10 407	8443	5406	14 317		
Population (€/capita)	104	230	118	245	270	298	292	155	214		

Farm data for EU Member States are for 2016 (Eurostat 2020), and for Russia from Mitchell and Baker (2019)

The myriad small farms in Poland and the Baltic states are in this sense a particular challenge, not only because persistently low incomes restricts their ability to finance investments for improving nutrient management, such as effective manure storage (Gorski et al. 2019), but also insufficient training in nutrient management. Available estimates suggest that nutrient surpluses are modest in those areas where many small farms are found (Svanbäck et al. 2019), but these are in all likelihood misleading since they are based on stylized calculations that do not take into account actual farm practices that tend to rely on ad-hoc storage of manure in heaps, inappropriate spreading techniques and poor seasonal timing of spreading. Conversely, foreign direct investment in large livestock facilities has introduced an industrial scale of agriculture to eastern BSR countries; in Poland, Lithuania and Latvia less than one per cent of holdings are rearing 40–50 per cent of all livestock (Table 1, Livestock). It remains to be verified whether these large livestock farms (> 100 livestock units) are on average more or less compliant with nutrient regulations than small farms, but it is to be expected that their huge volumes of manure are posing challenges that are amplified in a framework of lax enforcement.

The public support offered to farmers under the CAP, including national co-financing, differs among old and new Member States (Table 1, *Public Financial Support*). Farmers in Poland, Lithuania and Latvia are numerous and receive less average support, which is unfortunate considering the investment needs in, e.g. manure management. Farmers in the Baltic states receive far less support per ha agricultural land than farmers in Finland, Denmark and Germany, however, if financial support is considered per capita of population, the reverse is true. Polish farmers are in any case better off than Swedish farmers in terms of support per ha. These figures suggest that further financial support is not necessarily needed, but what is required seems rather to be *fair*, *stringent* and *targeted* support for nutrient management measures.

Overall, it needs to be kept in mind that behavioural change can be catalysed by demonstrating the economic implications of poor nutrient management, as the loss of nutrients is essentially a wasted resource. To improve nutrient management, farmers need to understand, for example, how manure mineralizes in soils and can substitute for costly mineral fertilizers, as well as manure’s value in maintaining soil health, which requires farmer education and advisory services.

The overriding challenges for the policy framework to overcome are therefore i) the continuing concentration of livestock resulting in extreme nutrient surpluses; ii) economic inequalities among farmers within and among BSR countries, particularly in Poland and the Baltic states; iii) gaps in nutrient management skills, iv) effective financing of abatement schemes; and v) the capacity for rolling out independent advisory services where they are currently lacking.

## Fully implement agreed-upon basic abatement measures

**Fig. 2:**
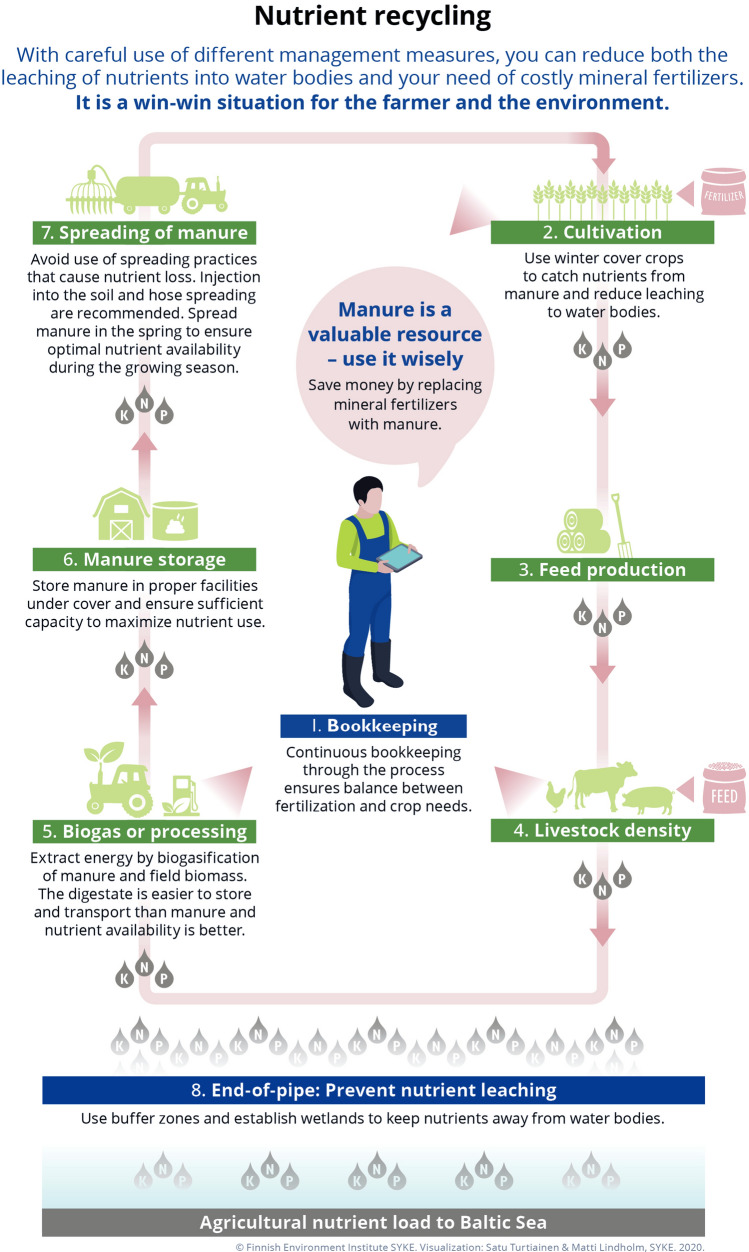
The role of BSAP abatement measures for improving nutrient recycling on farms and reducing nutrient emissions Source: The authors

In view of the extreme emissions risks associated with the concentration of livestock and resultant nutrient surpluses, BSR countries have agreed to basic nutrient abatement measures (Table S1). Different measures are required for reducing nutrient losses during the various stages of the nutrient cycle, which we overview in Fig. 2 numbered as Stages 1 to 8. Nitrogen is particularly labile, whereas P losses tend to occur as erratic runoff. Efforts to reduce emissions in one pathway can therefore increase losses in another, and thereby change the location, type and timing of environmental burdens (Hasler et al. 2019a). These emission risks can be minimized by containing and capturing the nutrients in livestock manure through judicious storage, timing and spreading of manure according to crop needs, thereby ensuring high manure-nutrient utilization by crops and enabling minimal application of mineral fertilizers (Webb et al. 2013).

Still, most countries have failed to integrate the full set of the Convention’s prescribed measures in national legislations, thereby undermining BSAP goals (see Thorsøe et al. (2021) for the research supporting this section). The most serious omissions are those relating to manure storage capacity and covers on storage tanks (Fig. 2, Stage 6), the manure-phosphorus application ceiling of 25 kg P/ha and shortcomings in manure application techniques (Stage 7), and maximum livestock densities (Stage 4). Furthermore, the failure of nutrient application ceilings to include mineral fertilizers exacerbates the potential for nutrient surpluses. The absence of a harmonized approach for accounting for the nutrient contents of manure is also a hinder to effective and comparable nutrient bookkeeping (Stage 1). Finally, the enforcement of regulations is inadequate and the penalties for breaching the rules are in most countries symbolic (Andersen et al. 2021).

### Ensure sufficient manure storage capacity and roofing

According to Jansson et al. (2019), improved manure spreading technologies in conjunction with upgrading of storage capacity could reduce the BSR’s nitrogen surplus by almost 18% (excluding Russia). Embargo periods banning the spreading of manure during periods with high risk for nutrient losses (Stage 7)—when land is bare, frozen, covered with snow or saturated with water—have been implemented in all countries, but are undermined by inadequate national prescriptions for manure storage capacity (Stage 6). Sufficient capacity is necessary to underpin the spreading of manure during the growing season (ideally in spring), but the Convention only prescribes a minimum of 6 months of capacity for liquid manure, which is inadequate to avoid post-harvest spreading. Most nutrient losses from fields occur during winter, when plant growth is minimal (Liu et al. 2018). Consequently, at least 8–9 months storage capacity is generally needed. Considering that until recently, four months of capacity sufficed for Polish farmers and that only 10–15% of farms in Lithuania and Russia have any storage capacity at all (ibid. Thorsøe et al. (2021), the weaknesses in this area are considerable and warrant immediate attention.

### Avoid ineffective technologies for spreading manure

Appropriate spreading technology is a fundamental abatement measure (Stage 7), but the conventional broadcast spreading technique is characterized by high nutrient losses. Trail hoses or injection into the soil ensure much higher utilization rates (Luostarinen 2013). Trail-hose and injection equipment are, however, more costly investments for farmers, which reduces the likelihood of their voluntary adoption if farmers do not appreciate the higher nutrient utilization rates or face financial constraints. Although the Convention requires that spreading is done ‘in a way that minimizes the risk of losses’, it does not feature an outright ban on broadcast spreading, hence it remains the predominant technology in most countries (Konrad et al. 2019).

Two additional obstacles prevent realization of the full abatement potential of improved manure management. First, the prevalence of solid manure on small and middle-sized farms as well as among large dairy farms where ~ 40% of the manure is solid (Tybirk et al. 2013, p. 9) prevents the use of these more effective application techniques. Second, the high costs of transporting manure from areas with a surplus to crop needs to those with a deficit tend to limit transport distances to only a few kilometres. High livestock densities are therefore in themselves a major obstacle to improve nutrient utilization in crop production. Despite the Convention requirement to avoid excess amounts of manure by prescribing maximum densities (Stage 4), only Estonia and Lithuania have such regulations in place.

### Create strong incentives to reduce extreme manure-nutrient surpluses

Considering that very large livestock installations represent hot-spots of pollution comparable to industrial sites or sewage treatment, even best-practice manure management will not suffice to avoid locally extreme manure surpluses since it is so costly to transport. Under these circumstances, manure processing, acidification or biogas fermentation (Stage 5) could be used to concentrate nutrients in less bulky form, lower transportation costs and promote broader utilization in crop production. Still, Tybirk et al. (2013) indicate that processing costs are high, corresponding to €0.35–1.50 per kg N, while the energy potential is limited due to the high water content and other complications.^2^ Consequently, these technical solutions are not likely to be attractive to farmers in the absence of stringent environmental policy.

It seems therefore necessary that livestock density regulations be strengthened to achieve acceptable nutrient utilization rates. Alternatively, a systematic penalty on nutrient surpluses (Stage 1) beyond a benchmark is a sure-fire way of creating economic incentives to reduce hot-spot surpluses, and promoting wider improvement and innovations in manure management (NIER 2014). Otherwise, it could also be considered to enrol large livestock farms in a nutrient cap-and-trade system that would equally well create incentives to reduce livestock densities or adopt technical solutions, which ever might be the least costly to reduce emissions (Hautakangas and Ollikainen 2019).

## Introduce performance-based payment schemes and charges

Reducing nutrient losses from fields in the BSR is additionally challenging, because the area of agricultural land is large and is heterogeneous in terms of societal, geographical and agricultural conditions (cf. Table 1). Land-use measures are therefore also necessary to achieve BSAP goals, but these are not supported through payment schemes in most countries and not well designed where used (ibid. Thorsøe et al. 2021). Especially buffer zones between fields and water bodies, and catch/cover crops are needed to capture surplus nutrients and underpin high plant utilization (Stage 2), while spatially targeted catchment-orientated measures such as creation and restoration of wetlands (Stage 8) can retain nutrients that, despite farmers’ best efforts, will still escape fields. Substantial public financial support is though available to complement environmental regulations,^3^ particularly to support investments in modern manure management technologies and for compensating farmers for the costs of voluntary land-use measures (see below on the EU’s role). However, the design of current land-use payment schemes (i.e. agri-environment-climate schemes) has severe weaknesses.

### Facilitating choice of cost-effective land-use measures

To be cost-effective, payments offered to farmers for land-use measures need to be optimized spatially in the choices of measures, and their area and placement (Shortle and Horan 2017). Higher abatement could therefore be achieved and at lower unit costs at the farm level if land-use measures were targeted to high-risk fields, but would need to be carefully designed to be perceived as fair and legitimate by farmers (Thorsøe et al. 2017).

Voluntary payment schemes to encourage farmers to adopt land-use measures are found mainly in the Nordic countries. These p*rescription*-*based* schemes typically offer farmers a payment for abatement measures on agricultural land, e.g. per hectare of a buffer zone.^4^ In contrast, there are no payment schemes for nutrient abatement that are *performance*-*based* in the sense that payment levels reflect the actual reduction in nutrient losses compared to a benchmark. The economics literature shows that prescription-based payment schemes are not cost-effective and hence not environmentally effective either (Ollikainen et al. 2019). The cost-effectiveness of a particular measure, in practice, will depend on spatial characteristics, e.g. in relation to the placement of a buffer zone primarily its width, proximity to water, and variety and density of vegetation, given the field’s soil type, gradient and climate zone (Jacobsen and Hansen 2016). Considering the heterogeneity of conditions in the BSR, the potential for prescription-based schemes to achieve cost-effective abatement of agricultural emissions to the Baltic Sea is absent.

The underlying reason for the inability of prescription-based payments to effectively contribute to reducing emissions can be traced to the inadequate linkage to actual environmental improvement. To achieve deep emissions reductions, while maintaining agricultural productivity, it is essential that farmers are rewarded for engaging in pollution abatement, which can be achieved by switching to some form of performance-based payments (Winsten 2009). The better the abatement effect, the higher the farmers’ payment when based on performance or results. Conversely, potential payments will be low where the effect is poor, thereby discouraging farmers from implementing measures where they have too little effect relative to the cost—and payment—to be a sensible use of farmers’ and taxpayers’ resources.

Furthermore, payments based on performance will provide incentives for innovations in farming practices that improve the effectiveness of existing measures and reduce costs over time. Given that farmers are motivated to a large extent by the pursuit of income from farming, payments based on performance will also promote cost-effective abatement, because it will be in the farmers’ interest to optimize the choice and placement of measures for nutrient abatement. Performance-based payments would also address the common criticism that todays’ (prescription-based) payments are too low to motivate many farmers to apply them, particularly on the most intensively farmed fields. Indeed, the potential for greater differentiation of payments has been found to improve the likelihood of uptake among farmers in Baltic Sea catchments (Hasler et al. 2019b).

### Realize performance-based abatement schemes using modelled results

A long-standing barrier for performance-based schemes has been the presumption that these require actual measurement of abatement effects at the field level (Burton and Schwarz 2013). However, if estimations can be made with precision other than by measuring, then these can function as a proxy for measurements (OECD 2010, p. 30). In fact for air pollution, estimations are often used where measurement would be too costly relative to the benefits, such as for smaller enterprises. Thus, if a farm’s emissions can be estimated with adequate precision, then performance-based schemes can be realized.

**Fig. 3:**
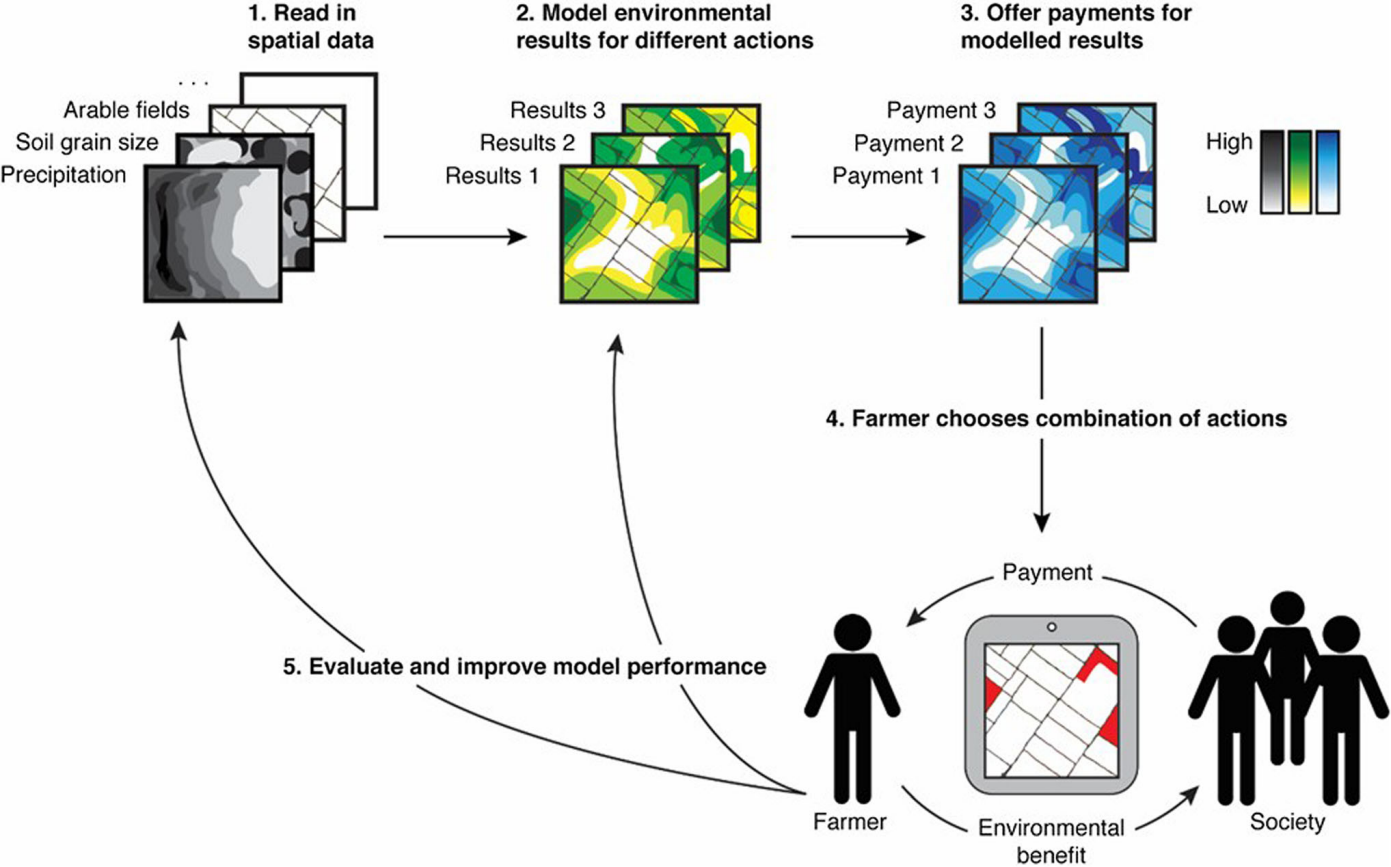
The concept of an agri-environmental payment by modelled results (PAMR) scheme Source: reproduced from Bartkowski et al. (2021) with permission from Elsevier

Bartkowski et al. (2021) develop the concept of an agri-environmental PAyment by Modelled Results (PAMR) scheme for estimating emissions, which is schematized in Fig. 3. The core idea is that instead of paying farmers for the area of a particular land-use measure, a PAMR scheme would employ a model to predict the results of farmers’ abatement choices, i.e. abatement effect. This scheme not only unites most of the advantages of a performance-based scheme based on measurements and the payment certainty for farmers of a prescription-based scheme, but also adds the potential to address trade-offs among multiple policy objectives and management for long-term environmental effects.

The current prescription-based approach stems from the early days of Baltic Sea governance, when data were limited and the biophysical relationships were poorly understood. Now, there exists sufficient science-based knowledge on the sources and impacts of measures on nutrient emissions that can be synthesized in models to inform policy and farmers’ decision-making (e.g. Hundecha et al. 2016; Reusch et al. 2018; Bauer et al. 2019). In particular, modern GIS software in combination with web-based applications make it feasible to take account of spatial variability, such as the proximity of a field to water bodies, soil quality, slope, climate, etc., and thereby realize a PAMR scheme (e.g. Talberth et al. 2015; Sidemo-Holm et al. 2018).

Such a scheme could be implemented in stages with increasing complexity (precision). Initially, one could focus on controlling farms’ nutrient surpluses. Eurostat routinely estimates surpluses by considering the aggregate nutrient outputs in farm products compared to inputs from fertilizers, biological fixation and atmospheric deposition (Eurostat 2013), which Blicher-Mathiesen et al. (2019) show can easily be applied at the farm level. The system could be based on, e.g. The Integrated Administration and Control System (IACS) that identifies the location of each parcel of agricultural land, its size and ownership to control agricultural policy payments in EU Member States. Given that payments today are contingent on farmers following good practices, deductions could then be made for surpluses beyond the reference level, thus differentiating according to how well farmers perform with the measures chosen.

In a second more advanced step it would be possible to use modelled predictions of annual emissions abatement and fully implement a PAMR scheme. Although a model might initially only be expected to identify the most desirable locations for payments and exclude the most undesirable, the approach has the potential to improve over time through validation and learning, particularly if perceived as a knowledge transfer system between scientists, farmers and society (Alavi and Leidner 2001). For instance, a model can be continually refined and improved through validation to field samples or recipient measurements (e.g. Andersen et al. 2014), interactions with stakeholders in the individual catchments (see Water Boards below) and the addition of new proven measures for farmers to choose from. The more advanced approach would not be too difficult to initiate in countries that have established adequate modelling frameworks, e.g. MONERIS in Germany and NLES in Denmark. In fact the recent scheme for public financial support for the construction of wetlands in Denmark relies on a combination of a bidding-procedure and estimates of the potential nitrogen reduction effects related to each site (MFVM 2019).

Finally, farmers are only likely to engage or increase their engagement in water quality management if it is in their interest to do so. One of the most important, but underestimated outcomes of using a payment approach based on performance, even if estimated, is that it increases farmers’ own understanding of the actions they can take and increases the freedom to make their own decisions (Winsten, 2009). Accordingly, a PAMR scheme has the potential to spur engagement and innovation in pollution abatement, which the experience in other sectors of performance-based environmental incentives demonstrates (Requate 2005).

### Strengthening the institutional framework

Eight of the nine BSR countries are members of the European Union, which gives their farmers access to considerable financial support through the Common Agricultural Policy (CAP). However, the EU is underutilized in pursuit of BSAP goals, in particular because HELCOM lacks the competence to act if a signatory is not in compliance (see Andersen et al. 2021 for the synthesis supporting this section). Further, the structure of public administration varies across the region, in particular local authorities in the Baltic states, Poland and Russia are largely deprived of own-staff and tax revenues, which limits their capacity to enforce water policy. Strong local authorities and independent advisory services are present mainly in the Nordic countries. Here we identify the potential for the EU and local institutions to complement each other, and the necessity of facilitating coordinated abatement among BSR countries to restore the Baltic Sea cost-effectively.

### Leveraging the EU for financial and institutional support

Rural Development Programmes (RDPs), which include agri-environmental and agri-climate measures, account for only 23% of CAP spending, while (ineffective) income support accounts for 73%. The European Court of Auditors also observes that water-quality-related measures have not been a priority for BSR countries’ RDPs, despite a total budget of 10 billion euro (ECA 2016, p. 39). Further, they observe a low level of interest among farmers for nutrient abatement, which is explained by the complexity of measures and, as also discussed above, the poor design and lack of payments for land-use measures. Somewhat timely, the post-2020 CAP reform is introducing the obligation for member states to direct substantial funding from income support to dedicated environmental payments in the form of *Eco*-*Schemes* that can be performance-based, which constitutes a major new capacity to reward farmers’ for nutrient abatement (Lampkin et al. 2020).

In terms of institutions, legal requirements for improving cross-sectoral coordination by involving environmental authorities in the disbursement schemes of RDPs should be obligatory, and a specific mechanism for linking to BSAP objectives established. This could be achieved by instituting formal coordination of RDPs with the river basin management planning cycle in BSR countries.

Despite the Nitrates Directive constituting a ‘statutory management requirement’ for receiving CAP support, the penalties for non-compliance are modest and not in proportion to the damages incurred (1–5% of area support, rising with recurring breaches), while the sheer complexity of penalty schemes causes considerable frustration among farmers. Introducing performance-oriented penalties (see above) would ensure that any imbalances between crop needs and nutrient surpluses would lead to commensurate reductions in support.

The EU’s Recommendation 2001/331/EC on environmental inspections is presently a non-binding act that applies to facilities that are subject to authorization under European law, whereby most farming activities are outside its scope. It seems timely to extend it to cover agricultural activities, particularly large livestock facilities, so that plans for inspections and the frequency of site visits are reported to the European Commission (2017). In addition to a common format for inspections and compliance activities, the monitoring, reporting and verification could be enhanced by establishing a regional-level inspection task-force similar to what exists in the context of the EU’s Common Fisheries Policy.^5^ In a similar way, an agricultural task-force would be responsible for verifying that national, regional and local authorities ensure compliance with statutory requirements.

Although the requirements of the EU’s Water Framework Directive (WFD) apply only to the coastal waters of the Baltic Sea, its preamble highlights the need for an effective and coherent water policy to take into account the vulnerability of ‘relatively closed seas’. By building on the EU’s legal framework and adapting it to better reflect the circumstances of the Baltic Sea, there exist opportunities for strengthening implementation of water policy. For example under the Marine Strategy Framework Directive, member states can request EU action when good environmental status cannot be achieved through domestic measures alone.

### Empowering stakeholders through local institutions

Inclusive forms of planning and stakeholder involvement are recognized as being crucial for successful design and implementation of water policy, because it ensures broad representation of local stakeholders, and allows for trust-building and collaboration (Morf et al. 2019). However, the cultural specificities of each BSR country tend to reflect deep-seated relations between institutions and individuals that are not easily changed, and for which nutrient management as a whole must adapt to become effective (ibid. Andersen et al. 2021).

The WFD prescribes the designation of river basin authorities to oversee integrated water management and river basin planning. These authorities are governed by Water Boards with representatives from local communities and associations. For example, in France the *Agences de l’eau* constitutes a separate layer of government, within an administrative culture otherwise characterized by a strong and centralized state (Scaduto 2016, pp. 32-38). These Water Boards have their own revenues and can set their own priorities according to the needs and circumstances in each catchment. In contrast, the river basin authorities of BSR countries are short of such resources vested in Water Boards, and thus of suitable power for supervising and coordinating management and abatement efforts in an effective, transparent and negotiated way. We suggest extending their capacity to act by granting them greater powers and funding (e.g. through the CAP’s RDP). In this way revenues could be directed not only to individual farmers, but also to building shared infrastructure, most urgently for collecting, processing and spreading manure.

Beyond governance institutions, the availability of competent advisory services is an essential basis for improving nutrient management (Pedersen et al. 2019). Distrust in the authorities is widespread among farmers, especially in the eastern BSR (Ptak et al. 2020), and too often commercial suppliers influence nutrient planning in the absence of independent advisors. However, such services are not well developed in eastern BSR countries, while privatizations restrict access for small farms due to the high costs (Labarthe and Laurent 2013). Considering the legacy of skepticism vis-à-vis the state in the eastern BSR, it will be vital that financial support, as provided through the EU, is directed towards farmer training and public-financed advisory services offering integrated agri-environmental knowledge (e.g. Sweden’s Catch the Nutrients service). In addition, rural multi-actor networks can also improve knowledge exchange, especially in a peer-to-peer environment where farmers learn from each other.

### Facilitate coordination of abatement among BSR countries

Finally, Andersson et al. (2021) conclude from their systematic review that the potential for restoring the Baltic Sea to good health is undermined by an abatement strategy, i.e. the revised BSAP (HELCOM 2013b) that is far more costly than necessary, and that is likely to be perceived as unfair by several countries; thereby undermining BSAP implementation. Facilitating coordination of abatement among BSR countries is therefore needed to allow flexibility in spatially allocating measures and thereby achieving the substantial cost savings—around €500 million annually according to the best estimate (Hyytiäinen and Ahlvik 2015)—that are possible compared to the current BSAP allocation of abatement among countries. To increase the willingness of countries to meet their commitments to reducing nutrient emissions in practice, fairness needs to be considered. As neither the BSAP nor the cost-effective solution can be considered fair according to standard criteria, side payments will also be needed to achieve a fairer distribution of abatement costs among BSR countries.

## Concluding remarks and recommendations

There is a pressing need to strengthen the policy framework for protecting the Baltic Sea from agricultural nutrient emissions, which still far exceed HELCOM targets. With respect to what BSR countries have agreed to do, we observed generally lax implementation of the Convention’s prescribed measures in most countries and with aggravating circumstances in many areas. In contrast to the Convention and BSAP, the European Union has economic, political and legal mandates to further implementation and compliance. By making more active use of the institutions of the EU for implementation of BSAP, it is possible to mobilize an economic and legal enforcement potential encompassing 90% of livestock and 95% of the agricultural land draining nutrients into the Baltic Sea. Most of the recommendations in this article are therefore as relevant to consider in the context of the EU as in the context of HELCOM, which we now elaborate on while noting that this does not preclude continued cooperation with Russia.

To begin with, even when prescribed measures have been formalized in national regulations, adequate inspections and penalties for breaking these are often missing, hence we also recommend a supra-national monitoring and compliance body to support national administrators funded by the EU.

Second, all BSR farmers apart from Russia benefit from the EU’s Common Agricultural Policy, but a large portion of this funding is being misspent contrary to specific CAP goals (Scown et al. 2020). Despite some targeted support for nutrient management (i.e. land-use measures) and efforts to ‘green’ the area-based income support, these efforts have not been effective (Hristov et al. 2020). Somewhat fortuitously, the ongoing CAP post-2020 reform delegates greater autonomy to Member States, by offering the potential to redistribute funding from general income support to performance-orientated environmental schemes through the new *Eco*-*Schemes*. Indeed, this seems necessary to meet the ambitious goals of the European Green Deal and Farm to Fork Strategy, which include achieving regional environmental goals.

Finally, there is a need for broadening financial support to promote better nutrient management and abatement measures. Agricultural advisors with expertise in nutrient planning have the potential to make a major contribution through communicating agronomic and environmental rationales to farmers, not the least because better nutrient management can generate win–win solutions. Independent advisory services and machine stations (e.g. who provide manure management services) should therefore also be eligible for CAP funding and not just farmers. Therefore, a formal coordination mechanism for Rural Development Programmes with river basin planning is recommended.

Over and above these top-down recommendations, complementary grass-roots institutions are needed for building trust with the farming community, and allowing for the integration of practice-based knowledge for improving the quality of nutrient management in the Baltic Sea catchment. In particular, we suggest instituting Water Boards, particularly in the eastern BSR countries, with own revenue sources as known elsewhere in Europe. Water Boards can provide multi-actor fora for building acceptance through consultation and decision-making involving the various stakeholders, particularly farmers.

Finally, a burgeoning governance challenge is climate change, which is expected to exacerbate nutrient emissions in coming decades (Reusch et al. 2018; Olesen et al. 2019), and additionally by changing socioeconomic factors such as land use, atmospheric deposition and wastewater emissions (Bartosova et al. 2019). These threats though are perhaps best considered in terms of the entire policy framework, such that policymakers consider that they are chasing a moving target and hence their choices of instruments must be capable of moving with it. We hope that our comprehensive stocktaking of the future governance needs will support policymakers in bridging the gap between the agreed abatement targets and measures, and what is actually being done to restore the Baltic Sea.

## Supplementary Information

Below is the link to the electronic supplementary material.

Electronic supplementary material 1 (PDF 265 kb)

## Footnotes

1 The signatory countries agreed to reduce their own annual inputs by 89,260 tonnes N and 14,374 tonnes P, while the remaining necessary reductions should come from shipping and non-signatory countries.

2 Nevertheless, Poulsen et al. (2019) report that separation could extend the break-even transport distance by up to 80 km for the manure P fraction in a Danish regulatory context.

3 Generally, farmers should only be paid to reduce pollution through voluntary payment schemes once they are complying with minimum environmental standards (OECD 2010). In this context the balancing principle of the EU’s Nitrates Directive and endorsed in the Convention Annex provides the minimum standard, thereby imposing a duty on farmers to balance nutrient inputs with crop needs by complying with the prescribed HELCOM measures.

4 We follow OECD (2010) terminology but a variety of synonymous terms are used for prescription-based measures: action- and measure-based or action-oriented payments/schemes, while performance-based payments/schemes are referred to as result-, outcome-, output-, or success-based/(oriented), or objective-driven or payment-by-result schemes (Bartkowski et al. 2021).

5 The European Fisheries Control Agency organizes joint control campaigns, where inspectors from different EU countries join forces, while the European Commission has its own inspectors, who can visit national authorities at any time to check they are implementing EU rules correctly. https://ec.europa.eu/fisheries/cfp/control/who_does_what.
