# Supplementary material for: Strengthening the policy framework to resolve lax implementation of the Baltic Sea Action Plan for agriculture
Source: Ambio. 2021 Jun 18;51(1):69–83. doi: 10.1007/s13280-021-01573-3 (PMC8651863; doi:10.1007/s13280-021-01573-3)
Supplement: Supplementary file 1 — Electronic supplementary material 1 (PDF 265 kb) [file 13280_2021_1573_MOESM1_ESM.pdf]

Ambio

ELECTRONIC SUPPLEMENTARY MATERIAL

This supplementary material has not been peer-reviewed.

Title: Strengthening the policy framework to resolve lax implementation of the Baltic Sea Action Plan for agriculture

Authors: Mark V. Brady, Mikael Skou Andersen, Anna Andersson, Emils Kilis, Sanna-Riikka Saarelae and Martin Hvarregaard Thorsøe

Contact: mark.brady@slu.se

Inventory of agricultural abatement measures prescribed by HELCOM

Table S1. Inventory of abatement measures as prescribed by HELCOM (2013) and the desirability of the available types of policy instruments for getting farmers to adopt them as judged by the authors (cf. Drevno 2016): (1) Capacity Building; (2) Regulations and Performance Standards which take into account Best Environmental Practice and Best Available Technology to reduce pollution from agricultural activities; (3) Nutrient Budgeting; (4) Voluntary Payment Schemes; (5) Investment Support; (6) Nutrient Taxes or Charges; and (7) Water Quality Trading.

**Description of measure**	**Desirability of different policy instruments to encourage adoption by farmers** [a]						
	**(1)**	**(2)**	**(3)**	**(4)**	**(5)**	**(6)**	**(7)**
**A. SOIL MANAGEMENT**							
a. Plant cover in winter		***					
b. Minimal cultivation systems				***			
c. Spring cultivation				***			
d. Catch crops				***			
e. Plough leys on sandy soils in autumn		***		**			
f. Controlled sub-surface drainage	**				***	*	
**B. FERTILISER AND MANURE MANAGEMENT**							
a. Fertilization plans and Nutrient balances	**		***			*	
b. Conversion to organic production	**			***		*	
c. Reduced fertilisation	***		*			**	
d. Manure application techniques			***		**	*	
e. Integration of fertiliser and manure nutrient supply	***		**			*	
f. Liming	***			**		*	
g. Avoid application of fertilisers and manure in high-risk areas	**	***				*	
h. Avoid application of fertilisers and manure in high-risk periods	**	***				*	
i. Manure and effluent storage capacity		***	*		**		
j. Transport manure to neighbouring farms			***			*	**
k. Slurry separation			***			*	**
l. Composting solid manure			***			*	**
m. Biogas production from manure and other agri-biomass			***		**	*	
n. Pelleting of manure			***		**	*	
o. Incineration of manure			***		**	*	
p. Maximum livestock density [b]		***	**			*	
q. Location and design of farm animal houses		***	**		*		
r. Maximum application rates for manure nutrients		***	**			*	
**C. ANIMAL FEEDING**							
a. Adopting phase feeding of livestock	***		**			*	

b. Reducing dietary nitrogen and phosphorus intakes	***		**			*	
c. Animal feed supplementation	***		**			*	
d. Wet feed and fermentation	na						
**D. FARM INFRASTRUCTURE**							
a. Establishment of wetlands				**	***		*
b. Buffer zones		**		***			*
c. Converting arable land to extensive grassland		**		***			*
**E. OTHER**							
a. Effective purification of runoff waters	na						
b. Ditch Filters and Dams		**			***	*	
c. Systematic on-farm Advisory Services	***	**				*	

Notes: a) According to authors' own ranking of the desirability of a particular policy instrument: *** Prime instrument; ** Complementary instrument; * Instrument indirectly encourages adoption of measure; blank implies least relevant; and 'na' that too little is known about the measure to make a judgement.

b) These measures are listed explicitly in Annex III Part 2 of the Helsinki Convention, but not in the detailed inventory of measures on which this table is based, i.e., HELCOM (2013).

References

Drevno, A. 2016. Policy tools for agricultural nonpoint source water pollution control in the US and EU. *Management of Environmental Quality: An International Journal* 27:106-123.

HELCOM. 2013. Revised Palette of measures for reducing phosphorus and nitrogen losses from agriculture. Accessed: 2020-03-23 https://helcom.fi/media/documents/Revised-palette-of-agri-environment-measures.pdf.
